# Effects of single and double mutations on the MYC promoter G-quadruplex using a custom G4 DNA microarray: conformational landscape and nearby guanine compensation

**DOI:** 10.1093/nar/gkag688

**Published:** 2026-07-15

**Authors:** Jonathan Dickerhoff, Desiree Tillo, Jinho Jang, Danzhou Yang, Charles Vinson

**Affiliations:** Borch Department of Medicinal Chemistry and Molecular Pharmacology, College of Pharmacy, Purdue University, West Lafayette, IN 47907, United States; Center for Cancer Research, National Cancer Institute, National Institutes of Health, Bethesda, MD 20892, United States; Borch Department of Medicinal Chemistry and Molecular Pharmacology, College of Pharmacy, Purdue University, West Lafayette, IN 47907, United States; Borch Department of Medicinal Chemistry and Molecular Pharmacology, College of Pharmacy, Purdue University, West Lafayette, IN 47907, United States; Purdue Institute for Cancer Research, West Lafayette, IN 47907, United States; James Tarpo Jr. and Margaret Tarpo Department of Chemistry, Purdue University, West Lafayette, IN 47907, United States; Center for Cancer Research, National Cancer Institute, National Institutes of Health, Bethesda, MD 20892, United States

## Abstract

Parallel DNA G-quadruplexes (G4) are key regulators of oncogene transcription. The MYC promoter G-quadruplex (MycG4) is a prototype parallel G4 and promising anticancer drug target. However, effects of DNA damage or mutations on their conformational landscape remain elusive. Systematic analysis of mutational effects is challenging because permutating G4-forming sequences creates a vast sequence space inaccessible to most experimental methods. Herein, using a custom G4-DNA microarray, we systematically examine all 2 145 possible single and double mutations of the MYC promoter G4. The results show no single or even double mutation completely prevents MYC G-quadruplex formation, emphasizing its exceptional robustness. Mutated MycG4 sequences can form G-quadruplexes with vacancies or bulges without the need to replace the damaged G-runs. Both length and position of a G-run determine its resilience against mutations. Intriguingly, our results reveal an efficient compensation mechanism for mutations involving nearby redundant G-residues to preserve G4 formation. Moreover, the most disruptive mutations involve two nonadjacent G-runs, which cannot be repaired by a single G-tract but can still be compensated by redundant guanines. These results provide critical insights into G-quadruplex folding, structural resilience, and damage tolerance, with implications for gene regulation and G4-targeted drug design.

## Graphical Abstract

**Graphical Abstract:**
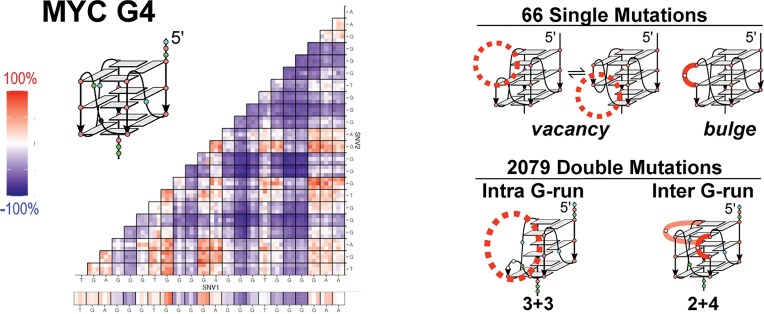

## Introduction

G-quadruplexes (G4) are four-stranded non-canonical secondary structures formed in single-stranded (ss) DNA or RNA sequences containing consecutive runs of guanosines. The G-quadruplex core consists of stacked G-tetrads, in which four guanines from different G-runs are connected by Hoogsteen hydrogen bonds. G-tetrad stacking is stabilized by cellular monovalent cations such as potassium and sodium [1]. In contrast to the linear and uniform duplex DNA, DNA G-quadruplexes are globularly folded and exhibit diverse structural features such as folding and loop conformations [2].

DNA G-quadruplexes are regulatory elements in cells. During transcription, double-stranded DNA is separated into two single strands (ss), and guanine-rich ssDNA spontaneously forms G-quadruplex structures [1]. These G-quadruplexes often coincide with transcription factor binding sites, the foci for recruiting transcriptional machinery, and regulate transcriptional activity [2–4]. The G-quadruplex formed in the MYC oncogene proximal promoter nuclease hypersensitivity element (NHE) III_1_ (MycG4) is one of the most prevalent G4-loci detected by the G4-binding antibody BG4 in immortal cells [5]. MycG4 functions as a transcriptional silencer [6, 7] and is a promising anticancer drug target [8–12].

**Figure 1.**
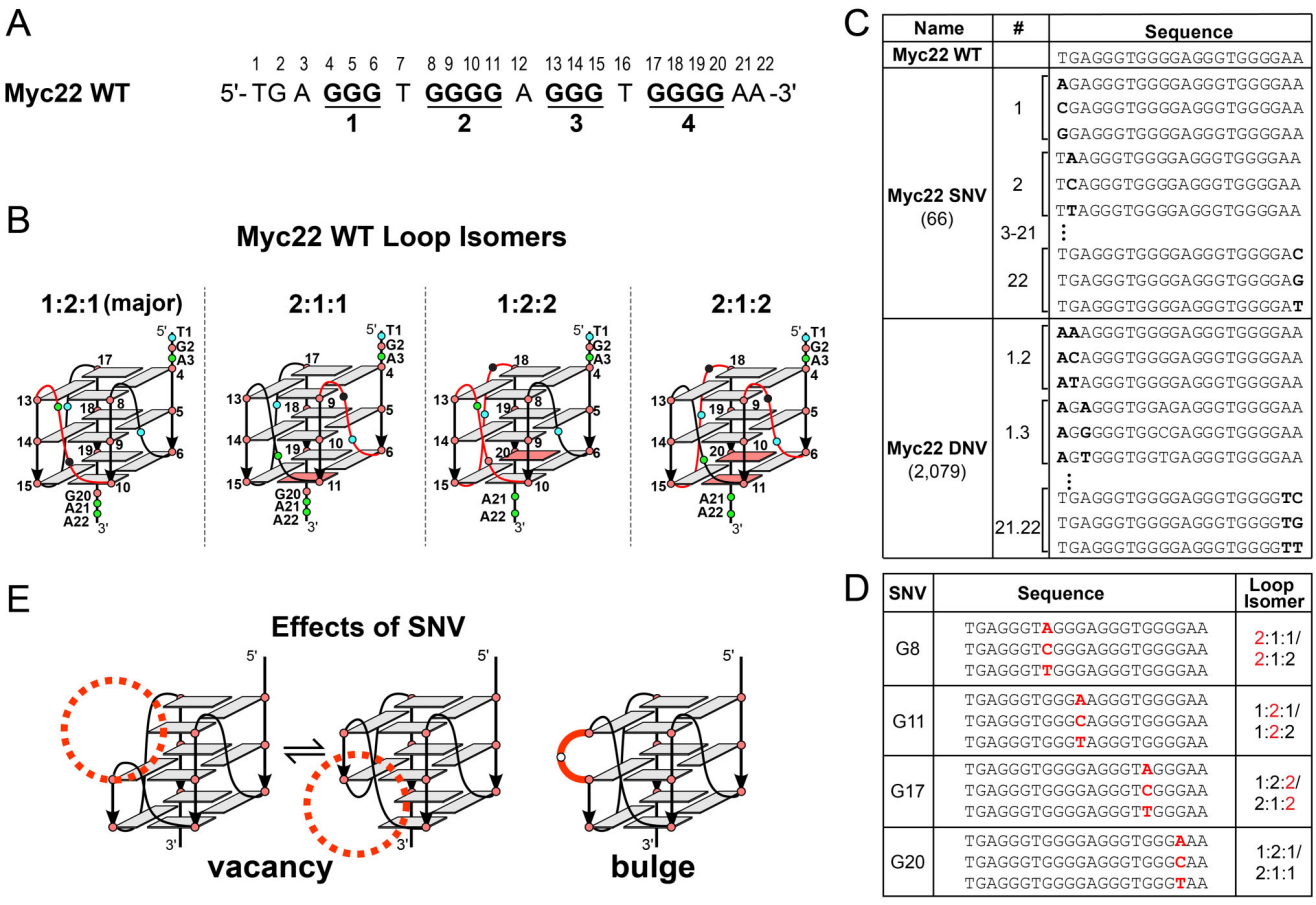
**A**) The MYC Promoter Myc22 WT sequence, which forms the major G-quadruplex. (**B**) Possible loop isomer conformations of the Myc22 WT G-quadruplex with the 1:2:1 loop isomer as the major form. Tetrad guanines that are not part of the major 1:2:1 isomer and the loops of 2-nt length are shown in red. (**C**) Summary of tested Myc22 WT, SNV, and DNV sequences. (**D**) SNVs tested to compare the loop isomers of Myc22. (**E**) Mutation of G-run residues can create a vacancy (shown by dashed circle) or bulge (shown by bolded arch) as destabilizing motifs.

The MYC promoter G-quadruplex is a parallel-stranded structure with three G-tetrads and a prominent model system for G-quadruplexes formed in human oncogene promoters [6, 13, 14]. An important feature of G-rich sequences in human promoters is guanine redundancy, characterized by more than four G-runs or G-runs containing more than the three guanines required to form a three-tetrad G-quadruplex. Such DNA sequences can form multiple co-existing G-quadruplex conformations, or loop isomers, by using different combinations of three guanines from each G-run to assemble the G-tetrad core [2]. For example, the MYC promoter sequence that forms the major G-quadruplex contains four consecutive G-runs consisting of 3, 4, 3, and 4 guanines, respectively (Myc22 WT, Fig. 1A) [6]. Since only three guanines from each 4G-run are required for the three-tetrad core, Myc22 WT can adopt four distinct parallel loop isomers (Fig. 1B). Importantly, all four isomers contribute to functional transcriptional control of the MYC oncogene [15]. We determined the high-resolution structure of the major MYC promoter G-quadruplex loop isomer 1:2:1, a three-tetrad, parallel-stranded conformation with loop sizes of 1, 2, and 1 nucleotides (Fig. 1B) [16].

DNA oxidative damage [17] and mutations in G4-forming regions can modulate gene expression and disease state [18, 19]. Many single nucleotide variants of the genomic Myc22 WT sequence have been reported in the Database of Single Nucleotide Polymorphisms [20]. However, the overall effects of mutations on the folding and conformational landscape of G-quadruplexes are largely unknown. Investigating such effects is challenging, because a G-quadruplex motif typically spans over 20 nucleotides, resulting in a large number of sequence permutations. This vast sequence space is challenging for conventional experimental methods, which generally assess one sequence at a time.

**Figure 2.**
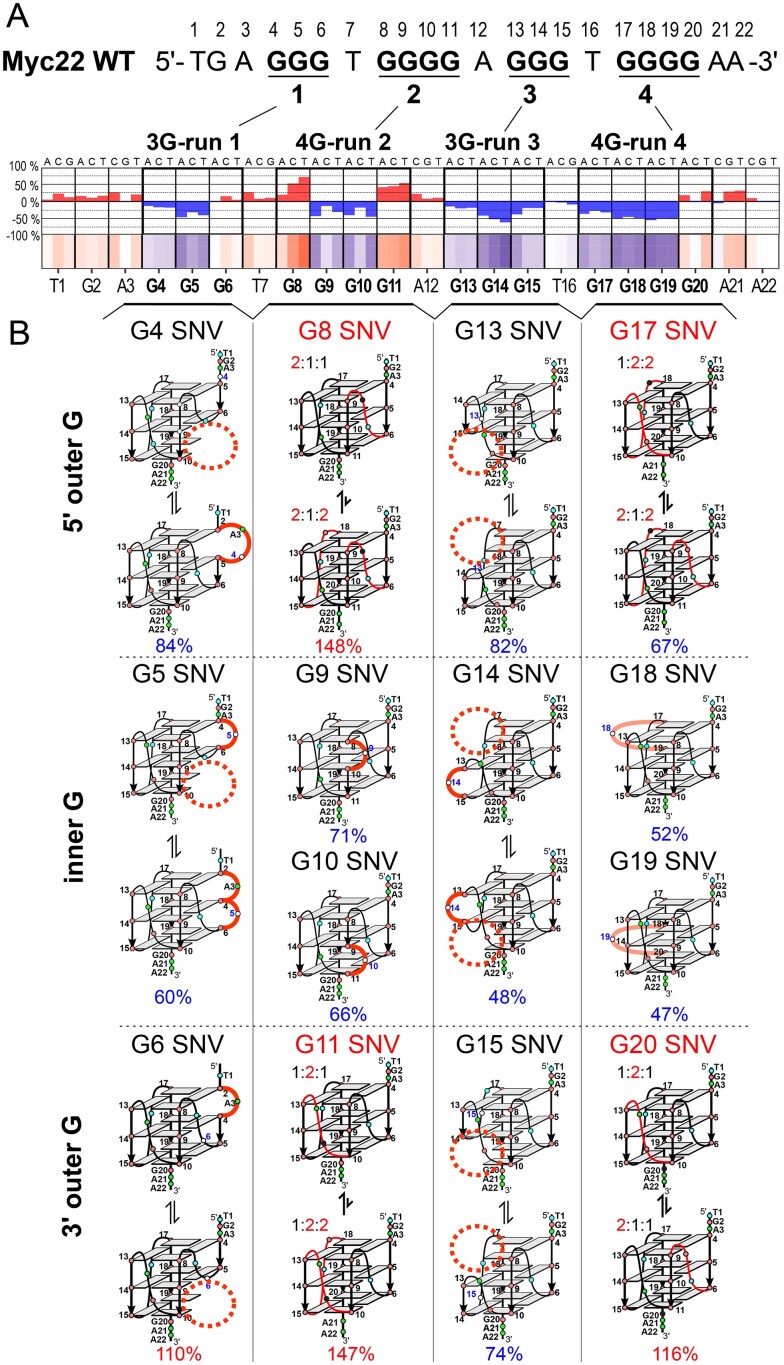
(**A**) MYC Promoter Myc22 WT sequence, which forms the major G-quadruplex (top), and plots of relative Cy5-PDS fluorescence for each of the 66 SNVs compared to Myc22 WT (bottom). For each nucleotide, the SNVs are always in the order ACG for thymine, ACT for guanine, AGT for cytosine, and CGT for adenine. The color indicates whether the Cy5-PDS binding to the SNV in general increases (red) or decreases (blue) compared to Myc22 WT. (**B**) Schematic structures of the most probable conformers for each G-run SNV. Mutated residues and destabilizing bulge and vacancy motifs are shown in red. The averaged fluorescence among the three SNVs of each position is listed.

To overcome this limitation, we developed a custom G4-DNA microarray containing approximately 15 000 different ssDNA sequences, primarily variants of known G4-forming sequences [21, 22]. Herein, we use this platform to systematically analyze the effects of all single and double mutations on the folding dynamics and conformational landscape of the major MYC promoter G-quadruplex. This dataset includes 2 145 DNA sequences, encompassing all 66 single nucleotide variants (SNVs, single mutations) and all 2 079 double nucleotide variants (DNVs, double mutations) of Myc22 WT (Fig. 1C and Supplementary Table S1). We employed pyridostatin, a G-quadruplex-specific ligand [23], conjugated to Cy5 (Cy5-PDS), to assess quadruplex formation via fluorescence. Differences in Cy5-PDS binding between variant sequences and Myc22 WT were shown as changes in fluorescence intensity, correlating with either enhanced or attenuated G-quadruplex formation (Fig. 2A). Furthermore, we evaluated the coupling effects between nucleotide positions in DNVs on G-quadruplex stability [24–26].

This comprehensive analysis of all possible SNVs and DNVs reveals how single and double mutations influence the folding and stability of the major MYC promoter G-quadruplex. Notably, no single or even double mutation was sufficient to completely abolish G-quadruplex formation. The results illustrate the contributions of individual G-runs and structural motifs to G-quadruplex integrity. Intriguingly, our findings demonstrate that mutated MycG4 sequences can still form stable G-quadruplexes with vacancies or bulges (Fig. 1E) [27–29], alongside canonical structures with continuous G-runs and complete tetrads, without the need to replace a damaged G-run. Furthermore, our findings reveal an effective and immediate compensation strategy through the use of nearby redundant guanines, instead of an intact G-tract ‘spare tire,’ to preserve G-quadruplex structure in the event of mutation or oxidative damage [30, 31].

Taken together, these results offer key insights into the folding, structural resilience, and damage tolerance of promoter G-quadruplexes, which are critical for understanding their function for gene regulation and G4-targeted drug design.

## Material and methods

### Microarray data

Cy5-PDS binding data were extracted from previously described custom Agilent G4 DNA microarray experiments [21]. This dataset includes all 66 single-nucleotide variants (SNVs) and all 2 079 double-nucleotide variants (DNVs) derived from the 22-nt Myc22 wild-type (WT) sequence, totaling 2 145 distinct Myc22 DNA sequences. To generate the SNVs, each position within the Myc22 WT sequence was individually substituted with the other three possible nucleotides. DNVs were generated by systematically permuting all possible nucleotide combinations at two distinct positions (Fig. 1C and Supplementary Table S1). Each 22-nt sequence (WT, SNV, or DNV) was capped at the 3′ end with an identical 38-nt single-stranded sequence covalently linked to the microarray glass slide surface. To induce G-quadruplex formation, the DNA microarrays were preincubated with KCl prior to exposure to Cy5-PDS. Binding was subsequently quantified via fluorescence microarray scanning as previously detailed [21]. For robust quantification, each unique DNA sequence was printed in 11 independent replicate probes across the microarray, from which the median fluorescence intensity was computed. The raw and processed microarray datasets are publicly available in the NCBI GEO database under accession number GSE133368.

### Circular dichroism (CD) spectroscopy

Individual Myc22 sequences (WT, SNVs, and DNVs) utilized for CD and NMR studies were synthesized via solid-phase synthesis as previously described [32]. The oligonucleotides were dissolved at a final concentration of 5 µM in a pH 7.0 buffer containing varying concentrations of K^+^: 25 mM (18.75 mM KCl and 6.25 mM potassium phosphate), 50 mM (37.5 mM KCl and 12.5 mM potassium phosphate), or 100 mM (75 mM KCl and 25 mM potassium phosphate). CD spectra at 25 °C and melting experiments were measured with a JASCO-1100 spectropolarimeter (JASCO, Inc.) using a 10 mm quartz cuvette. Parameters for spectra acquisition were a 1 nm data pitch, 1 nm bandwidth, 2 s response time, a scanning speed of 100 nm/min, and 5 accumulations. The buffer spectrum was subtracted from all spectra for blank correction. CD melting experiments were performed by measuring ellipticity at 263 nm for each sequence every 1 °C between 20–95 °C with a heating rate of 2 °C /min and a digital integration time of 2 s. Melting temperatures (*T*_m_) were determined with the Spectra Manager Version 2 software (JASCO, Inc.) using the second derivative of the melting curves.

### Nuclear magnetic resonance (NMR) spectroscopy

Synthesized Myc22 DNA sequences were dissolved to 90 µM in 25 mM K^+^ buffer (18.75 mM KCl + 6.25 mM potassium phosphate), pH 7, with 90% H_2_O and 10% D_2_O. 1D ^1^H NMR spectra were collected at 25 °C using a Bruker NEO 500–1 spectrometer equipped with a Prodigy cryoprobe. Excitation sculpting was employed for water suppression, and ^1^H chemical shift was referenced directly based on the water signal relative to 4,4-dimethyl-4-silapentane-1-sulfonic acid (DSS). Spectra were processed using Bruker TopSpin 3.6.

### Calculation of expected binding

Expected binding for each DNV was determined by multiplying the ratios of binding compared to WT for each SNV (SNV1/WT x SNV2/WT). For example, if binding of the first SNV is 1/2 WT and the second SNV is 1/3 WT, the expected binding of the DNV is 1/6 WT.

## Results

### Effects of single mutations (SNVs) on G-quadruplex formation of Myc22 WT

We first examined the effects of the 66 Myc22 SNVs on G-quadruplex stability (Fig. 2A). Microarray experiments can be reliably conducted at a near-physiologically relevant K^+^ concentration of 100 mM to form only intramolecular (monomeric) structures, since each ssDNA is an extended 60-nt oligomer containing either the Myc22 WT or its SNVs and DNVs, and is immobilized on a solid base, preventing intermolecular interactions with neighboring DNA molecules.

The wild-type MYC promoter sequence, Myc22 WT, contains two 3G-runs (G-run 1 and 3) and two 4G-runs (G-run 2 and 4) (Fig. 1A). For a 4G-run, mutation of the first or last G (Fig. 1D) creates a 3G-run that can form a standard G-quadruplex with continuous G-runs and complete tetrads (Fig. 1B). All mutations on 3G-runs, as well as a mutation of an inner G on 4G-runs, disrupt a continuous G-run in the MYC G-quadruplex (Fig. 1E). Mutation of an inner G of a 4G-run can induce the formation of a bulge containing the mutated residue (Fig. 1E) [27], while a mutation on a 3G-run may result in a vacancy G-quadruplex (vG4), in which one tetrad is incomplete and consists of only three guanines (Fig. 1E) [28, 29]. Surprisingly, the observed fluorescence of most inner SNVs changes only modestly, by less than 25% compared to Myc22 WT (Fig. 2A), indicating that mutated MycG4 sequences can form stable G-quadruplexes with vacancies or bulges (Fig. 1E), without the need to replace a damaged G-run. The most disruptive SNV is the inner G of the 3G-run 3, i.e., the G14 SNV (48% of the Myc22 WT) (Fig. 2A). Intriguingly, the SNVs in the other 3G-run 1 had much smaller negative effects. Unexpectedly, the inner SNVs of the 4G-run 4, including G18 (52%) and G19 (47%), showed strong negative effects (Fig. 2A). It should be noted that fluorescence intensity provides only an approximate correlation with G4 stability, and small discrepancies likely reflect experimental variability rather than meaningful differences in structural folding. Therefore, we emphasize overall trends rather than minor variations in absolute values in the following sections. Additionally, potential variations in ligand accessibility across different sequences cannot be fully excluded. Specifically, the G8 or G11 SNVs exhibit higher fluorescence than the Myc22 WT; this is likely due to reduced structural dynamics caused by the G8 or G11 mutation [33, 34], which subsequently leads to increased Cy5-PDS binding.

### Circular dichroism (CD) and NMR analysis of individual MYC mutants to validate microarray findings

To confirm insights obtained from the large-scale microarray data, we conducted CD spectroscopic, CD melting, and NMR experiments on individual mutated Myc22 DNA sequences. As MycG4 is highly thermostable, most spectroscopic experiments were performed at 25 mM K^+^ to reliably obtain melting temperatures that can be compared, as well as to prevent higher-order structure formation.

**Figure 3.**
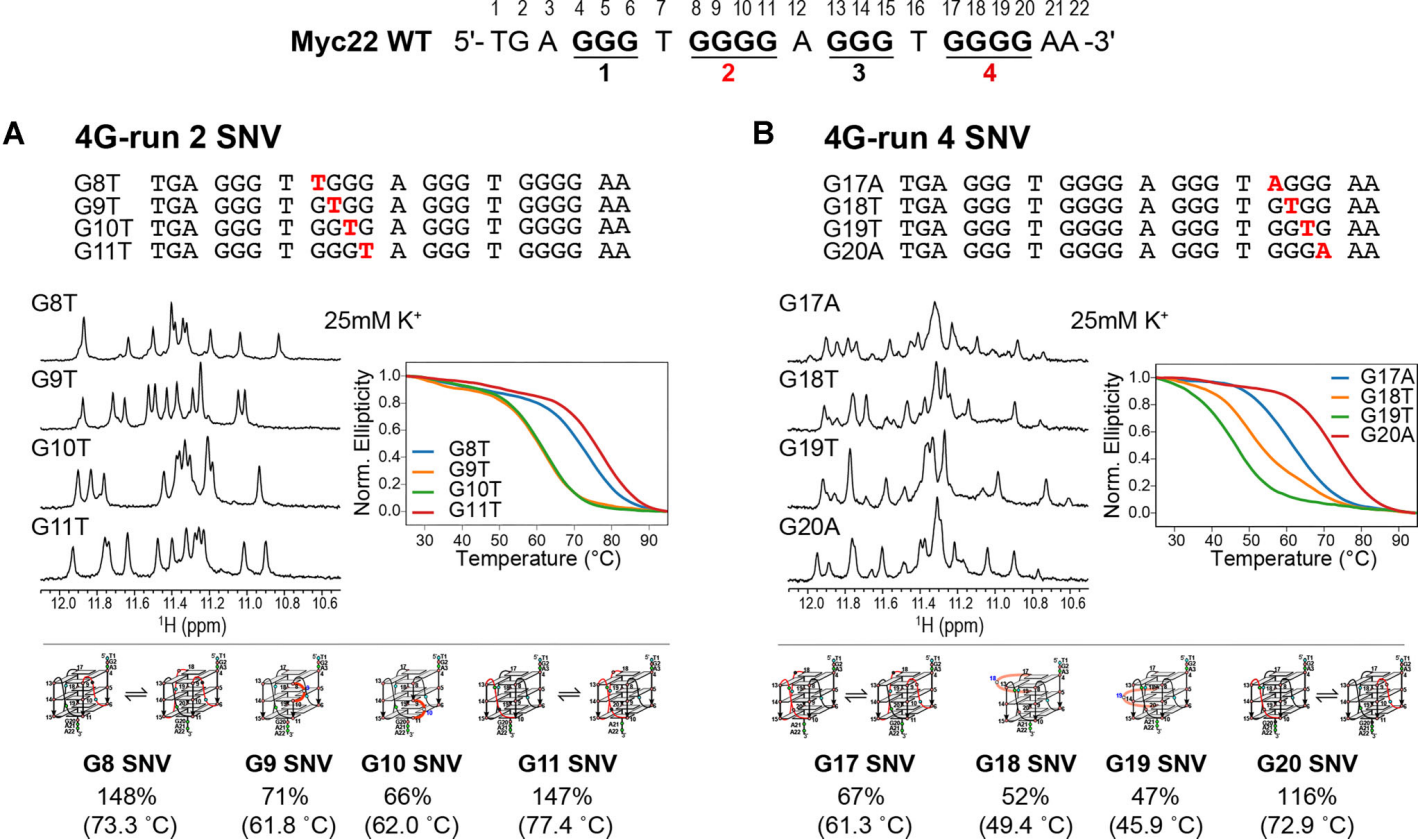
1D NMR imino proton region and CD melting for selected SNVs of the two 4G-runs with sequences shown. (**A**) Selected SNVs of 4G-run 2. (**B**) Selected SNVs of 4G-run 4. Most probable conformers are shown at the bottom together with the averaged fluorescence among the three SNVs of each position and the melting temperature for the tested sequence.

**Figure 4.**
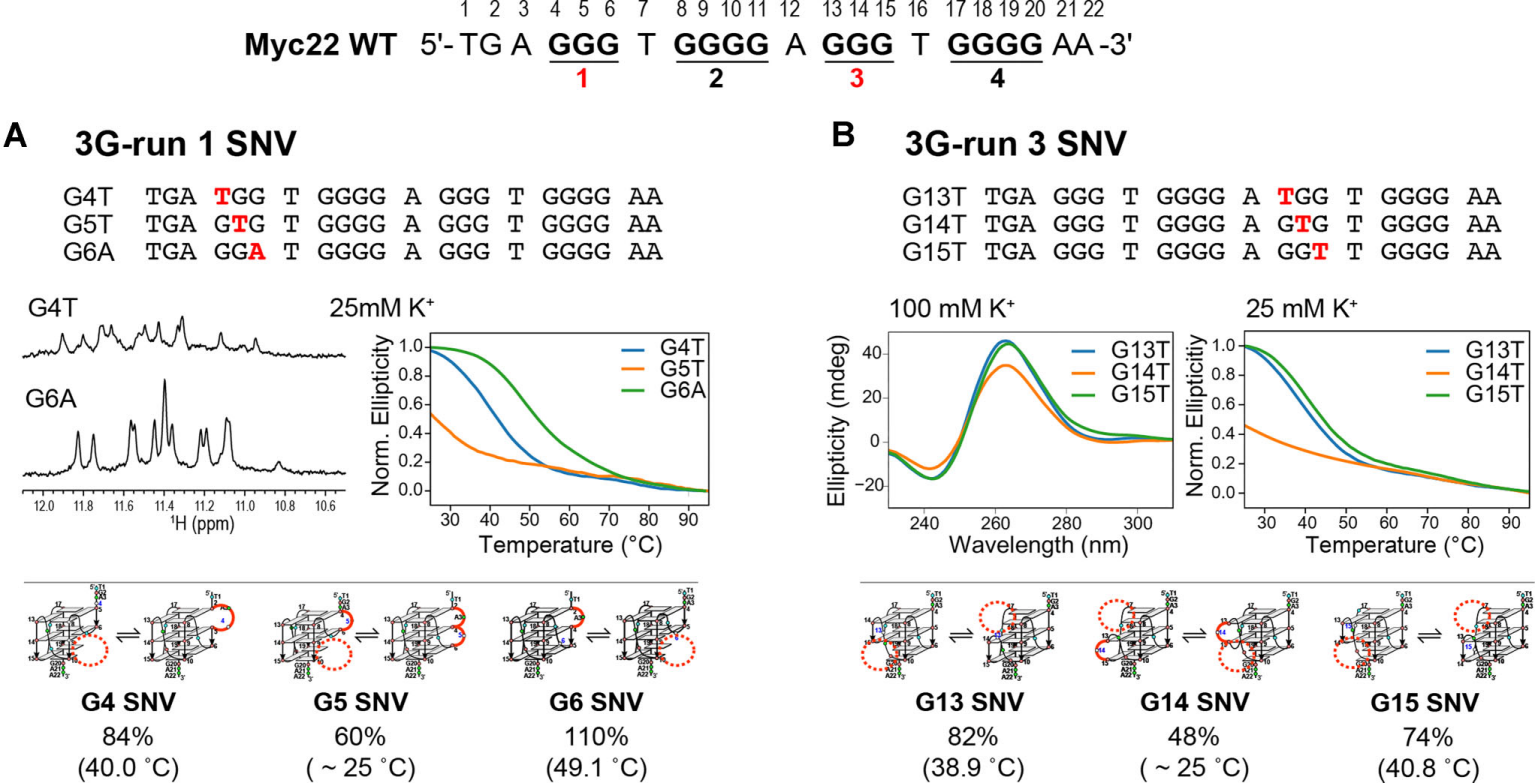
1D NMR and CD data for selected SNVs of the two 3G-runs with sequences shown on top. (**A**) 1D NMR imino proton region and CD melting for selected SNVs of 3G-run 1. (**B**) CD spectra and CD melting for selected SNVs of 3G-run 3. Most probable conformers are shown at the bottom together with the averaged fluorescence among the three SNVs of each position and the melting temperature for the tested sequence.

The CD spectroscopic experiments on SNV mutation sequences for all G-runs show a strong positive signal at about 260 nm, indicating a parallel folding structure (Figs 3, 4, and S2). Based on this, we proposed the most probable folding structures for each of the G-run SNVs, assuming a parallel G-quadruplex with potential vacancy or bulge formation (Fig. 2B).

### Distinct behaviors of the two 4G–runs despite both forming canonical loop isomers upon outer–residue mutation

SNVs in 4G-runs can lead to two possible outcomes. SNVs of the outer Gs remove G redundancy, but a continuous 3G-run remains (Fig. 1D), isolating one of the MycG4 loop isomers, which are distinctly folded canonical parallel G-quadruplexes with three complete G-tetrads (Fig. 1B) [15]. SNVs of the inner Gs of the two 4G-runs break the G-run continuity and lead to a less stable G-quadruplex with a bulge.

For the 4G-run 2, the outer SNVs (G11 or G8) lead to the loop isomers 1:2:1(/2) or 2:1:1(/2) (Fig. 2B), which show similar but clearly higher fluorescence than the inner SNVs (G9 or G10) (Fig. 2A). However, the 4G-run 4 behaves differently, in which two outer Gs (G17 and G20) are not equivalent. G20 SNVs exhibit markedly stronger fluorescence than G17 SNVs (Fig. 2A), indicating G17 is strongly preferred over G20 for incorporation into the G-tetrad core (Fig. 2B). This inequality has been observed in previous studies using individual DNA sequences [15, 34–36] and makes 4G-run 4 resemble a 3G-run.

Notably, the G8 or G11 SNVs exhibit increased fluorescence, indicating greater Cy5-PDS binding compared to Myc22 WT (Fig. 2A). This effect likely arises from the loss of structural dynamics in which G8 and G11 alternate between the G-tetrad core and loop, an energetically favorable process [33, 36] that would reduce Cy5-PDS residence at the binding site. Consistent with this dynamic effect, the G8 and G11 SNVs show large variability in fluorescence intensity (Supplementary Fig. S1).

### Inner–G mutation within a 4G–run induces a broken–strand G–quadruplex with a 1–nt bulge

SNVs of the inner Gs (G9, G10, G18, or G19) of the two 4G-runs of Myc22 WT disrupt the G-run continuity and may induce a 1-nt bulge to maintain a complete G-tetrad core (Fig. 2B), which is less stable than WT MycG4 with clearly decreased fluorescence (Fig. 2A). The two 4G-runs again behave differently for their inner SNVs. In 4G-run 2, SNV mutations of inner G-residues (G9, G10) exhibit similarly reduced fluorescence. In contrast, inner SNVs (G18 or G19) in the 4G-run 4 exhibit more pronounced and different (non-equivalent) reduction in fluorescence (Fig. 2A). This result suggests that inner SNVs in the 4G-run 2 (G9 or G10 SNVs) can be compensated by outer G8 and G11 in a bulged G-quadruplex (Fig. 2B). However, the inner SNVs (G18 or G19) in 4G-run 4 cannot be effectively compensated, likely due to the unfavored involvement of G20 in the G-tetrad core (Fig. 2B).

### CD and NMR analyses of individual 4G-run SNV sequences support microarray results

CD and NMR data of individual Myc22 SNV DNA sequences (Fig. 3) confirm the insights obtained from the large-scale microarray data. A mutation in a 4G-run either isolates one of the MycG4 loop isomers in the case of outer-G SNVs or induces a less stable G-quadruplex with a bulge for inner-G SNVs. Consistently, CD melting experiments reveal higher thermal stability for outer-G SNVs (G11 in 4G-run 2; G17 and G20 in 4G-run 4) compared to inner SNVs (e.g. G9T/G10T in 4G-run 2; G18T/G19T in 4G-run 4) (Fig. 3). For example, in 4G-run 2, G11T has a melting temperature of 77 °C, whereas inner SNVs G9T and G10T melt at ∼62 °C, 15 °C lower (Fig. 3A).

In 1D NMR spectra, the G-tetrad imino peaks between 10 and 12 ppm can monitor G-quadruplex formation because they are isolated and usually well resolved. Observation of 12 imino signals shows the formation of a G-quadruplex with three complete G-tetrads. For example, 12 imino protons are observed for the loop isomers 1:2:1(/2) and 2:1:1(/2) formed by the outer G11 and G8 SNVs in 4G-run 2, respectively (Fig. 3A). An inner mutation within a 4G-run is proposed to generate a bulged structure with three complete G-tetrads rather than a vacancy (Fig. 2B). Intriguingly, this is supported by the 1D NMR spectra: the imino region (10–12 ppm) shows 12 well-resolved peaks for G9T and G10T, consistent with three complete G-tetrads without a vacancy (Fig. 3A).

Microarray data show differences between 4G-run 4 and 4G-run 2: outer Gs (G17 and G20) in 4G-run 4 are not equivalent, with G17 strongly preferred in the G-tetrad core. CD melting data mirror the microarray results, with thermal stability decreasing as G20 (*T*_m_ 72.9 °C) > G17 (*T*_m_ 61.3 °C) > G18 (*T*_m_ 49.4 °C) > G19 (*T*_m_ 45.9 °C) (Fig. 3B). G20T’s *T*_m_ (∼73 °C) is similar to G11T, whereas G17T is 12 °C lower, emphasizing G20’s unfavorable involvement in the MycG4 core. Inner SNV G18T and G19T sequences exhibit markedly lower *T*_m_ values (49.4 °C and 46 °C) compared to G9T and G10T (62 °C), indicating inefficient compensation by outer-Gs in 4G-run 4 due to G20’s unfavorable involvement in the G-tetrad core.

NMR further underscores these differences. All G-run 4 SNV sequences retain an intact 4G-run 2 and display mixtures of two species in the 1D NMR spectra, indicating dynamic positional shifting of G8 or G11 within 4G-run 2 (Fig.  3B). In contrast, the G9T and G10T SNV sequences, which retain an intact 4G-run 4, exhibit only a single dominant species in the 1D NMR spectra, reflecting the absence of dynamic shifting of G17 or G20 within 4G-run 4 (Fig. 3A). The lack of dynamic shifting further supports the highly unfavorable incorporation of G20 into the G-tetrad core and is consistent with the trends observed in both the microarray binding and CD melting experiments.

### 3G–run SNVs form vacancy– or bulged–G–quadruplexes, but their impact on 3G–run 1 is markedly milder due to compensation by a nearby redundant guanine

For the two 3G-runs 1 and 3, mutations of the first or last G-residue (outer G) induce a vG4 with an incomplete 5′- or 3′-tetrad (Fig. 1E). For example, in the 3G-run 3, SNVs at either of the outer guanines (G13 or G15) produce a mixture of two vG4s with vacancies in either the 5′- or 3′-tetrad (Fig. 2B). Consequently, no significant difference in stability is observed between the G13 and G15 SNVs.

Intriguingly, SNVs in the 3G–run 1 exhibit noticeably smaller disruptive effects compared to those in the 3G–run 3 (Fig. 2A). Particularly, a clear difference is observed between the G6 SNVs and G15 SNVs, the 3′-end residues of the two 3G-runs. Unlike G15, G6 SNVs show minimal disruption (Fig. 2A). This suggests a compensatory effect of the redundant proximal G2 residue, which can fill in under formation of a 1-nt bulge to complete the 5′-tetrad, as proposed in Fig. 2B for G6 SNVs. The G4 SNVs are more disruptive than G6 SNVs, likely because the G2 compensation would induce a 2-nt bulge, which is less stable than 1-nt bulges (Fig. 2B, G4 SNVs) [27].

Mutating the inner G of a 3G-run (G5 or G14) induces a vacancy in the central tetrad of the MycG4, which is energetically unfavorable because it generates a central cavity resulting in the loss of both stacking interactions and Hoogsteen hydrogen bonds. Therefore, it is more likely that the structure adopts a bulged G–quadruplex, in which the mutated residue forms a bulge, and the vacancy is shifted to a terminal tetrad, thereby reducing the disruptive effect of a central cavity (Fig. 2B, G5 and G14 SNVs). Accordingly, for the inner G14 SNVs in 3G–run 3, the most likely structural outcome is the formation of a 1–nt bulge accompanied by relocation of the vacancy to a terminal G–tetrad (Fig. 2B). The presence of these two disruptive motifs together explains the markedly reduced fluorescence signal (48%). Again, a less disruptive effect is observed for G5 SNVs in the 3G-run 1 (Fig. 2A), likely because the nearby G2 can compensate for the mutated G5: an alternative structure with two bulges of 1 nt length may form, recruiting G2 to participate in the G-tetrad core and thereby retaining all Hoogsteen hydrogen bonds and full tetrad stacking (Fig. 2B, G5 SNV, bottom).

### NMR and CD analyses validate the proposed folding model and compensation by the proximal redundant guanine

For the 3G-run 1, the microarray data reveal an intriguing and important insight: the nearby redundant G2 in the 5′-flanking region can compensate for mutational damage. In agreement with the microarray results, both the G4 and G6 SNV show 12 imino proton peaks in the 1D NMR spectra (Fig. 4A), with the G6 SNV exhibiting superior spectral quality. The presence of 12 imino peaks indicates formation of a G-quadruplex with three complete G-tetrads without vacancies, supporting the proposed bulged structure in which G2 fills into the tetrad core as the major conformation (Fig. 4A bottom). With G2 fill-in, the 1-nt bulge of the G6 SNV is more favorable than the 2-nt bulge of the G4 SNV, as reflected by the superior NMR spectral quality of G6 SNV. This difference in structural stability is further supported by CD melting experiments, with thermal stability of G6 > G4 > G5 (Fig. 4A), fully consistent with the microarray results (Fig. 2A). These findings reveal an effective and immediate compensation strategy through the use of nearby redundant guanines, rather than complete unfolding and refolding involving an intact G-tract “spare tire”, to preserve G-quadruplex structure under mutation or oxidative damage [30, 31].

In contrast, the 3G-run 3 outer SNVs form vG4s and lack a nearby redundant guanine for compensation. Consequently, G13T and G15T SNVs exhibit similar melting temperatures but are less stable than the G6 SNV (Fig. 4B).

For inner G5T and G14T SNVs, the combination of two bulges or a bulge plus a vacancy in their proposed structures severely compromises G4 stability, as seen in both microarray (Fig. 2A) and CD melting data (Fig. 4B). G14 SNVs are less stable than G5 SNVs, again suggesting favorable G2 fill-in for G5 SNVs. At lower salt concentration (25 mM K^+^), G5 and G14 SNVs appear to be less stable at room temperature; however, at 100 mM K^+^, a stable G-quadruplex forms, as indicated by the strong positive CD band near 260 nm (Fig. 4B).

Overall, the CD and NMR data for individual SNVs are in full agreement with the microarray findings. Together, these complementary data demonstrate that microarray fluorescence reflects G–quadruplex stability and that the proposed folding structures provide a reasonable explanation for the observed behaviors. This convergence of data strengthens confidence in the subsequent analysis of the more complex DNV results.

### Overview of DNV data on G-quadruplex formation of Myc22 WT

**Figure 5.**
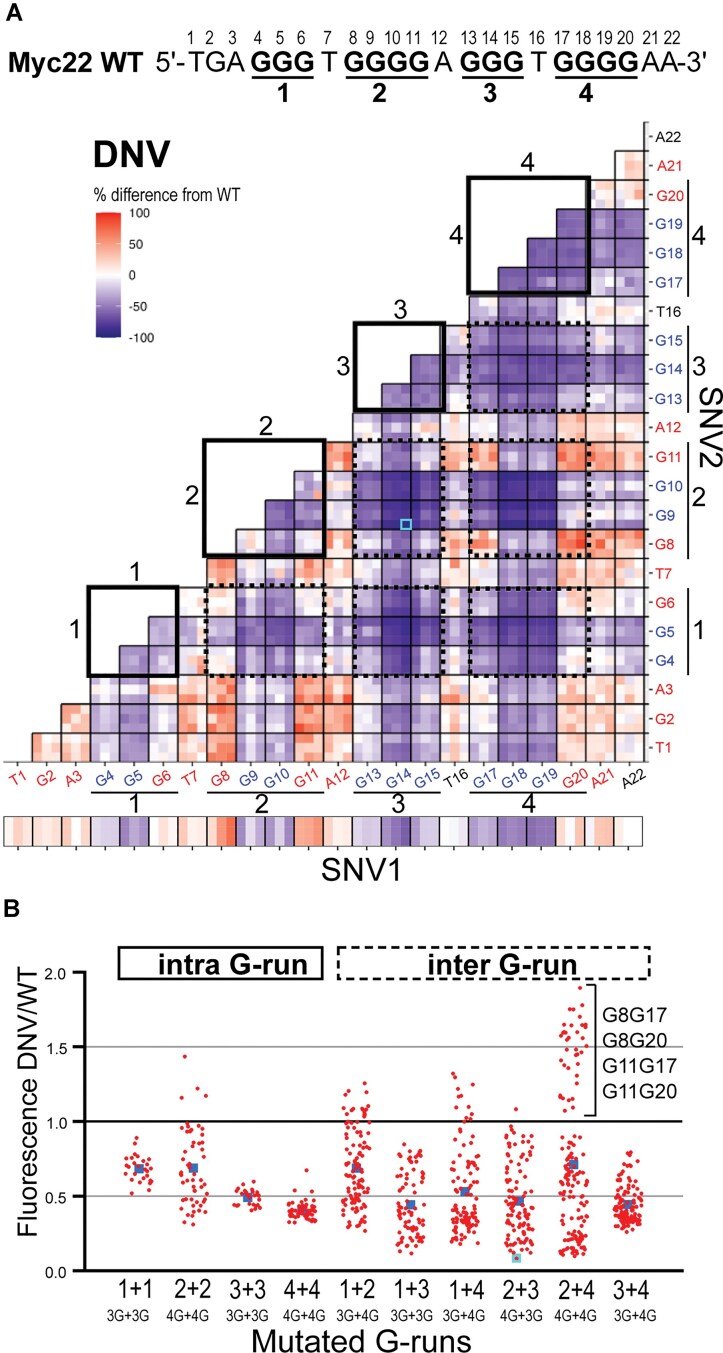
**A**) Heat map of Cy5-PDS binding to the 2079 DNVs of Myc22 WT, with each variant presented uniquely, with the Myc22 WT sequence representing the major G-quadruplex from the MYC promoter shown on top. DNVs are identified by the SNVs along both the horizontal and vertical axes. For each nucleotide, the SNVs are always in the order ACG for thymine, ACT for guanine, AGT for cytosine, and CGT for adenine. DNVs occurring within a guanine run and between guanine runs are outlined with solid and dashed boxes, respectively. The DNV with the lowest fluorescence is marked by a cyan box. (**B**) Plot of the fluorescence relative to WT for each DNV only involving G mutations separated by the involved G-runs. The average of each group is marked by a blue box, and the DNV with the lowest fluorescence is labeled with a cyan box.

We next examined the DNV data of 2 079 double nucleotide mutation sequences representing all possible combinations for Myc22 WT (Fig. 1C). The DNV data are presented as a heat map where each square (pixel) represents one of the 2 079 DNVs, with the two corresponding SNVs shown on the horizontal and vertical axes (Fig. 5A). Clusters of weaker binding are observed for DNVs where both mutations are in G-runs (rectangles), which either involve mutations in the same G-runs (intra G-run, solid line) or different G-runs (inter G-run, dashed line). To further analyze the effects of DNVs, the changes in fluorescence compared to Myc22 WT were plotted for inter G-run and intra G-run DNVs (Fig. 5B).

DNVs can result in stronger destabilization than SNVs. Greatest destabilization is observed for DNV mutations of inner G-run residues (Fig. 5A). Notably, the observed fluorescence of most DNVs changes by less than 50% compared to Myc22 WT, and no double mutation can completely prevent the formation of G-quadruplex structures. Even the most negatively affected DNV combination G9G18, has an average fluorescence about 1000-fold higher than the weakest binding observed in the non-G4-forming sequences among the 15 000 ssDNAs [21].

### Intra G-run DNVs involving the last two G-runs or inner Gs are more disruptive

**Figure 6.**
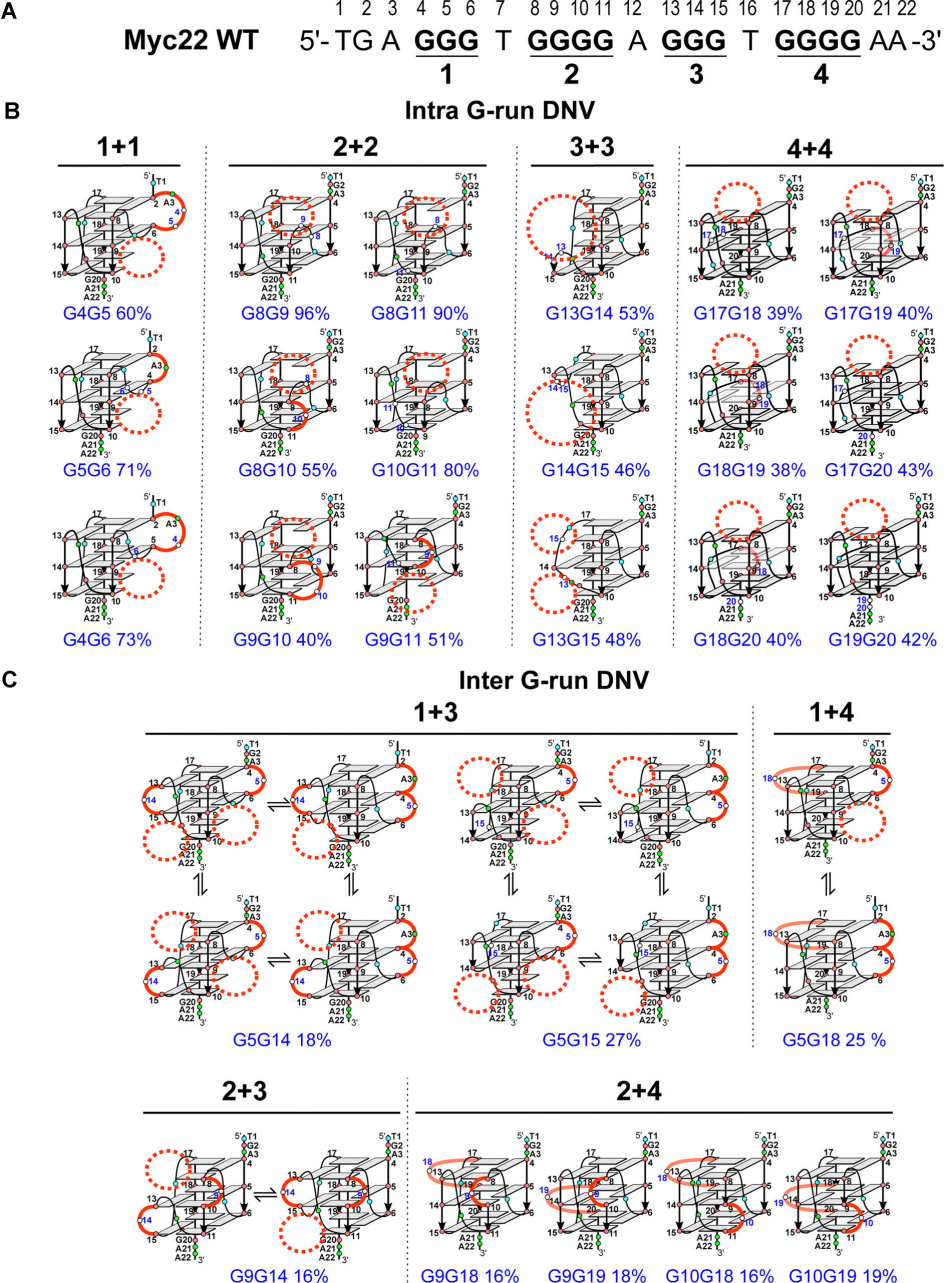
(**A**) MYC Promoter Myc22 WT sequence, which forms the major G-quadruplex. (**B**) Potential schematic structures of intra G-run DNVs with the averaged fluorescence of each position listed below. Mutated residues and disruptive bulge and vacancy motifs are highlighted for schematic structures. (**C**) Potential schematic structures of eight selected sequences representing inter G-run DNVs with the lowest fluorescence, with the averaged fluorescence of each position listed below. Mutated residues and disruptive bulge and vacancy motifs are highlighted for schematic structures.

Averaged fluorescence for intra G-run DNVs in general decreases from G-run 1–4, with lowest values observed for the last two G-runs (Fig. 5B), like the SNV data but with more profound effects. In general, intra-G-run DNVs involving the last two G-runs are more disruptive, as shown by lower fluorescence (Fig. 5B). Probable folding structures were created for the intra DNVs (Fig. 6B).

A consistently lower fluorescence is observed for intra DNVs in 3G-run 3 (∼50% of the Myc22 WT fluorescence) (Fig. 5B), which is expected as these DNVs will disturb two of the three tetrads (Fig. 6B, 3 + 3). However, the intra DNVs of the first 3G-run 1 are significantly less affected (Fig. 5B). The observed fluorescence of most disruptive intra DNVs of 3G-run 1 is ∼70% compared to Myc22 WT despite two mutations in a 3G-run. This again suggests a likely compensation by the 5′-proximal G2 as observed for the G-run 1 SNVs (Figs 2B and 6B, 1 + 1).

Intra-DNVs in the last 4G-run 4 show overall pronounced negative effects (Fig. 5B and Table 1), despite having a spare G residue which seems to lack compensatory potential. This result also supports the idea that G20 is unlikely to be involved in the G-tetrad-core so that the 3′-terminal 4G-run behaves more like a 3G-run.

**Table 1.** Most disruptive intra-G-run and inter-G-run DNVs with the ratio of observed fluorescence for DNV over WT

**Intra DNV** ^a^		**G-runs**	**DNV/WT**
G4 + G5	intra	1 + 1	0.602
G9 + G10	intra	2 + 2	0.405
G14 + G15	intra	3 + 3	0.464
G18 + G19	intra	4 + 4	0.378
**Inter DNV** ^b^		**G-runs**	**DNV/WT**
G5 + G10	inter	1 + 2	0.328
G5 + G14	inter	1 + 3	0.181
G5 + G19	inter	1 + 4	0.250
G9 + G14	inter	2 + 3	0.162
G9 + G18	inter	2 + 4	0.155
G14 + G19	inter	3 + 4	0.319
**All Inter DNV < 30%** ^c^		**G-runs**	**DNV/WT**
G9 + G18	inter	2 + 4	0.155
G9 + G14	inter	2 + 3	0.162
G10 + G18	inter	2 + 4	0.164
G9 + G19	inter	2 + 4	0.176
G5 + G14	inter	1 + 3	0.181
G10 + G19	inter	2 + 4	0.193
G10 + G14	inter	2 + 3	0.210
G5 + G19	inter	1 + 4	0.250
G5 + G18	inter	1 + 4	0.253
G9 + G15	inter	2 + 3	0.257
G10 + G15	inter	2 + 3	0.257
G5 + G15	inter	1 + 3	0.271
G4 + G14	inter	1 + 3	0.288
G6 + G14	inter	1 + 3	0.291

^a^Lowest intra-G-run DNV combination for every G-run.

^b^Lowest inter-G-run DNV combination for every G-run combination.

^c^All inter-G-run DNV combinations with < 30% of Myc22 WT fluorescence.

Intra-DNVs in 4G-run 2 show a notably higher variation than other G-runs due to its spare G residues (Fig. 5B). DNV mutations of both outer Gs or two adjacent mutations at the beginning or end of 4G-run 2, such as G8G9, G8G11, and G10G11, retain 80–90% of G-quadruplex formation compared to Myc22 WT, indicating a stable G-quadruplex with a well-tolerated vacancy (Fig. 6B, 2 + 2). In contrast, the DNVs involving two non-adjacent G or two adjacent inner guanines G9 and G10 (G8G10, G9G11, G9G10) create a discontinuous G-run and are strongly disfavored (Table 1). These inner DNVs create both a vacancy and a bulge, causing low stability and thus fluorescence, while the 2-nt bulge formed in G9G10 DNVs further reduces stability (Fig. 6B, 2 + 2).

### Inter G-run DNVs are more disruptive than intra G-run DNVs

All six groups of inter DNVs show high fluorescence variability (Fig. 5B). Potential schematic structures for the eight most disruptive DNV sequences, which have < 30% of Myc22 WT fluorescence, were proposed to better understand their strong disruption (Fig. 6C). Two to four disruptive vacancy or bulge motifs are found among the conformers. The mutation of 3G-runs in the 1 + 3, 1 + 4, and 2 + 3 subsets enables co-existing structures. Either a vacancy can move back and forth between the 5′- or 3′-tetrad, like for 3G-run 3, or the 5′-flanking G2 residue can compensate for a mutation to prevent vacancy formation in case of 3G-run 1. Consequently, the inter-DNVs G5G14 and G5G15 with two mutated 3G-runs (1 + 3) have the most complex folding equilibria with four conformations and multiple disruptive motifs (Fig. 6C). Table 1 summarizes the most disruptive inter-G-run DNVs.

**Figure 7.**
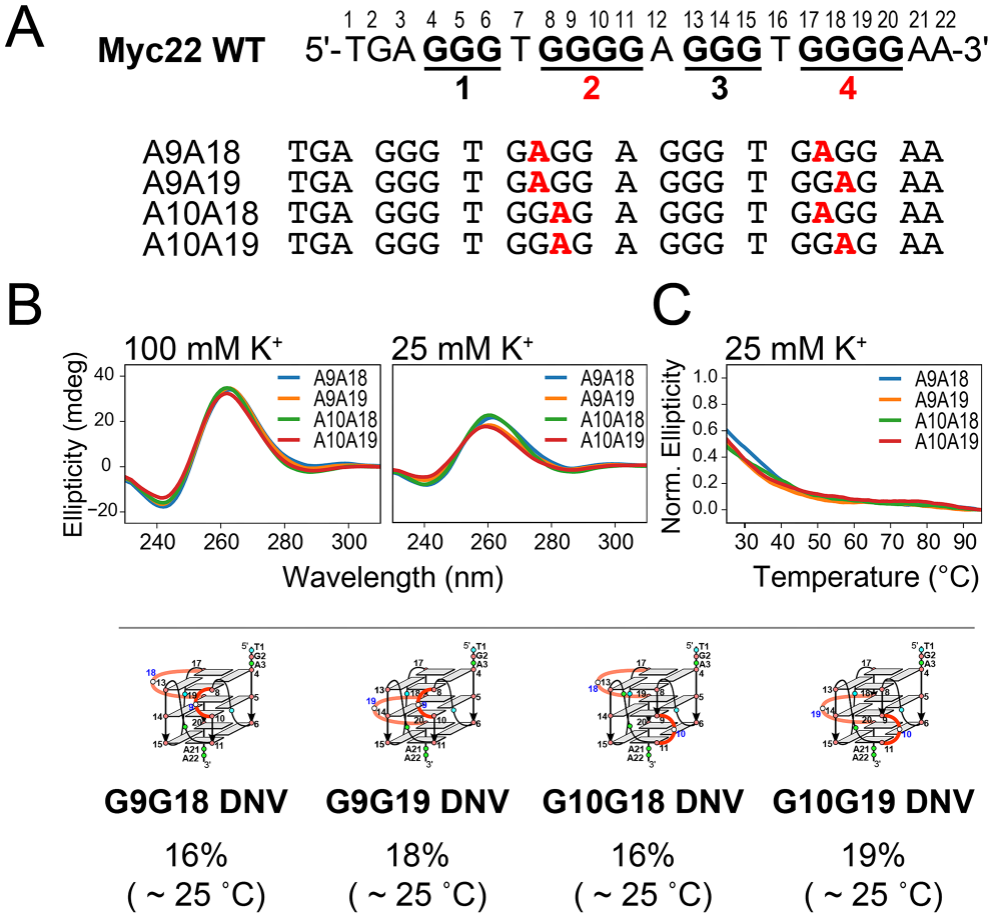
(**A**) Representative 2 + 4 DNV sequences with inner G-mutations. (**B**) CD spectra and (**C**) CD melting curves for the selected 2 + 4 DNV sequences. Most probable conformers are shown at the bottom together with the averaged fluorescence from microarray data and the CD melting temperatures for the tested DNV sequences.

DNVs involving the inner G mutations of 4G-run 2 are the most disruptive, as seen in 2 + 4 and 2 + 3 DNVs (Table 1, Inter). The most negatively affected DNV combination appears to be G9G18 DNV (Fig. 6C, 2 + 4). The inner G mutation of a 4G-run, such as G9 or G18 in G9G18 DNV, may induce a bulge or a vacancy if the bulged conformation is unstable. We conducted CD spectroscopic and CD melting experiments for the representative 2 + 4 DNV sequences with two inner G-mutations, including the G9G18, G9G19, G10G18, and G10G19 DNVs (Fig. 7A). The CD spectra confirmed that the individual 2 + 4 DNV sequences still form parallel G-quadruplex structures despite two G-run mutations (Fig. 7B), supporting the microarray data. The CD melting data showed similar low melting temperatures for the representative 2 + 4 DNV sequences with inner G-mutations (Fig. 7C).

A small subset of inter-DNVs involving G8 or G11 shows higher fluorescence than Myc22 WT (Fig. 5A, 2 + 4 rectangle with G8G17, G8G20, G11G17, G11G20). Each of these DNVs has four continuous 3G-runs and can form a standard three-tetrad G-quadruplex. The increase in bound Cy5-PDS is likely due to reduced folding dynamics as observed for SNVs (Fig. 2A). Interestingly, inter DNVs involving G-run 1 + 2 also show notable variability and less disruption (Fig. 5B), again suggesting the compensation potential of the nearby 5′ G2.

### Coupling between SNVs: Observed vs. Expected Cy5-PDS binding of the DNVs

**Figure 8.**
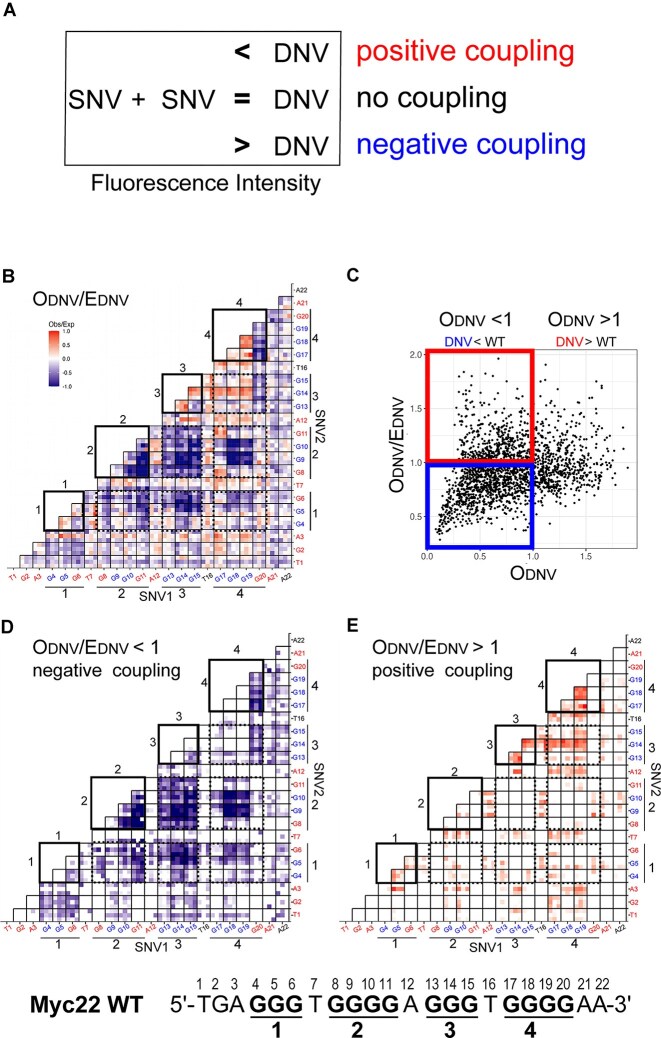
(**A**) Conceptual model illustrating the effects of positive and negative coupling between mutations on the observed fluorescence intensity. (**B**) Heat map of Observed/Expected (O_DNV_/E_DNV_) ratio for Cy5-PDS binding to Myc22 WT DNVs analogous to Figure 3A. (**C**) Scatter plot for Myc22 WT DNVs comparing observed (O_DNV_) fluorescent intensity and O_DNV_/E_DNV_. D-E) Heat maps showing Myc22 WT DNVs with O_DNV _< 1. D) O_DNV_/E_DNV _< 1 and (**E**) O_DNV_/E_DNV _> 1. For all heatmaps, DNVs occurring within the same G-run and in different G-runs are outlined by solid and dashed boxes, respectively. MYC Promoter Myc22 WT sequence, which forms the major G-quadruplex, is shown at the bottom.

The SNV and DNV data for the 2 145 Myc22 WT-based sequences allow analysis of coupling between nucleotides in the G4 structure. Coupling describes a synergistic effect on the G-quadruplex stability that is larger than the sum of the underlying SNVs (Fig. 8A). We examined the observed (O_DNV_) and expected (E_DNV_) binding of all 2 079 DNVs of Myc22 WT (Fig. 8B). O_DNV_ is the ratio of the DNV fluorescence to Myc22 WT fluorescence, while E_DNV_ is calculated by multiplying the O_SNV_ of the two SNVs in the DNV (O_SNV_ is the ratio of SNV fluorescence to Myc22 WT fluorescence). If O_DNV_/E_DNV_ is greater than 1, this indicates positive coupling, whereas if O_DNV_/E_DNV_ is less than 1, this indicates negative coupling. We further classified coupling by plotting O_DNV_/E_DNV_ against O_DNV_ (Fig. 8C). A small portion of DNVs show higher fluorescence than wild-type Myc22 WT (O_DNV _> 1, Fig. 8C right quadrants), mostly involving G8 or G11, where the increase in fluorescence is likely due to reduced folding dynamics but not increased G-quadruplex formation because Myc22 WT is completely folded into a G-quadruplex under the used conditions. Therefore, DNVs with O_DNV _> 1 are not meaningful and heatmaps were only generated for DNVs with O_DNV _< 1 (Fig. 8D-E) from the two left quadrants of the scatter graph Fig. 8C. This analysis mimics the double mutant cycle analysis used to measure the energetic interaction between two amino acids in a protein [24–26, 37], but with the caveat that for DNA we actually look at differences relative to the wild type sequence instead of a “neutral” alanine mutation.

### Strong negative coupling for 2 + 3 and 2 + 4 inter-DNV subsets

A main category comprises inter-DNVs with O_DNV_/E_DNV _< 1 (Fig. 8C (blue rectangle) and D). Strong negative couplings for inter-DNVs are clustered on the 2 + 3 and 2 + 4 subsets. In most cases, both of the SNVs are disruptive, and the two mutations enhance the disruptive effect (Figs 5 and 6C). This highlights the central role of 4G-run 2 in MycG4 folding and stability due to its central location. Moreover, it indicates the synergistic detrimental effects of the combined mutations in the non-adjacent 4G-runs 2 and 4.

### Negative coupling for intra-DNVs of the two 3G-runs

Negative coupling was observed in the two 3G-runs for intra-DNVs with two outer Gs’ mutation (Fig. 8D, diagonal boxes for G-run 1 and 3). Here, two of three tetrads are disrupted in the DNVs, whereas the corresponding SNVs disrupt only one external G-tetrad and show only slightly decreased O_SNV_, or even an increase for G6 SNVs due to G2 compensation (Fig. 8D).

### DNVs that show positive coupling

Intra-DNVs of the last two G-runs and inter-DNVs from the 3 + 4 subset show O_DNV_/E_DNV _> 1 (Fig. 8C (red rectangle) and E). These positive couplings involve SNVs, such as G14 and G17-G19, which have the lowest fluorescence. However, the double mutations cannot fully destroy the G-quadruplex formation, so this indicates a limit of possible disruption is reached.

In addition, the heat map with positive coupling (Fig. 8E) reveals several examples of flanking or loop residues whose mutation to G positively compensates a G-run mutation. For example, the flanking residue A3G SNV compensates for the inner G5 mutation in the adjacent 3G-run 1 and shows a positive coupling effect. Compensatory potential for SNVs in adjacent G-runs can also be seen for the three loop residues T7, A12, and T16. Such compensatory effects are limited to the 3G-runs and not observed for 4G-runs, probably due to the intrinsic potential of the spare G in 4G-runs to compensate single mutations.

## Discussion

G-quadruplexes are key regulatory elements in promoter regions of many oncogenes, and the structure formed in the MYC promoter is a prototype of parallel G-quadruplexes. Using custom G4-DNA microarrays, we have systematically analyzed the set of 2 145 DNA sequences, including all possible single (SNVs) and double-nucleotide (DNVs) mutations of the major MYC promoter G-quadruplex on its conformational landscape and folding dynamics.

Mutation of any inner Gs within 3G- or 4G-runs reduces G-quadruplex stability, whereas mutation of the outer Gs of a 4G-run does not affect G-quadruplex formation. While 3G- and 4G-runs contribute differently to stability, the impact seems to be more dependent on the position of a G-run within the sequence. Mutations on the second and third G-runs are of particular importance for MycG4 stability, likely due to their central location.

Significantly, no single or double mutations can completely prevent G-quadruplex formation. Even the most disruptive double mutation forms a parallel G-quadruplex with an average fluorescence ∼1000-fold higher than the weakest observed in the negative non-G4 DNA controls on the microarray. The MYC promoter G-quadruplex forms under conditions of dynamic supercoiling associated with elevated MYC transcription, where it functions as a negative regulatory element by blocking activating transcription factors and/or recruiting transcriptional repressors. The remarkable structural resilience observed in our study suggests that this regulatory mechanism may remain operative even under conditions of oxidative damage or accumulated mutations, which are frequently encountered in cancer genomes. In particular, our results indicate that G quadruplex structures can still form despite single or even double-nucleotide variations, thereby preserving the capacity for G quadruplex–based transcriptional control and protein interactions.

Intriguingly, sequences with single or double mutations still form stable G-quadruplexes with vacancies or bulges without the immediate need to replace the damaged G-run. Particularly, our findings suggest a compensation strategy for DNA damage through the recruitment of a nearby redundant guanine. In promoter G4 sequences, a fifth G-tract has been proposed as a ‘spare-tire’ to replace a damaged or mutated G-run and maintain G-quadruplex integrity [30]. However, such replacement requires unfolding of the existing G-quadruplex, whereas utilizing nearby redundant guanines may offer a more kinetically favorable and structurally accessible mechanism for preserving G-quadruplex formation. The 5′-flanking G2 residue exemplifies this strategy, readily compensating for mutations in the first 3G-run. Furthermore, the most disruptive double mutations occur in non-adjacent G-runs, which cannot be rescued by a single spare G-run but may be compensated by nearby redundant guanines.

## Supplementary Material

gkag688_Supplemental_Files

## Data Availability

The microarray data are available at the NCBI GEO database (https://www.ncbi.nlm.nih.gov/geo/) under accession no. GSE133368.
