# Supplementary material for: Effects of single and double mutations on the MYC promoter G-quadruplex using a custom G4 DNA microarray: conformational landscape and nearby guanine compensation
Source: Nucleic Acids Res. 2026 Jul 15;54(13):gkag688. doi: 10.1093/nar/gkag688 (PMC13369326; doi:10.1093/nar/gkag688)
Supplement: gkag688_Supplemental_Files [file gkag688_supplemental_files.zip › SI_revised.pdf]

Supporting Information

Effects of Single and Double Mutations on the MYC Promoter G-Quadruplex Using a Custom DNA Microarray: Conformational Landscape and Nearby Guanine Compensation

Jonathan Dickerhoff, Desiree Tillo, Jinho Jang, Danzhou Yang*, and Charles Vinson*

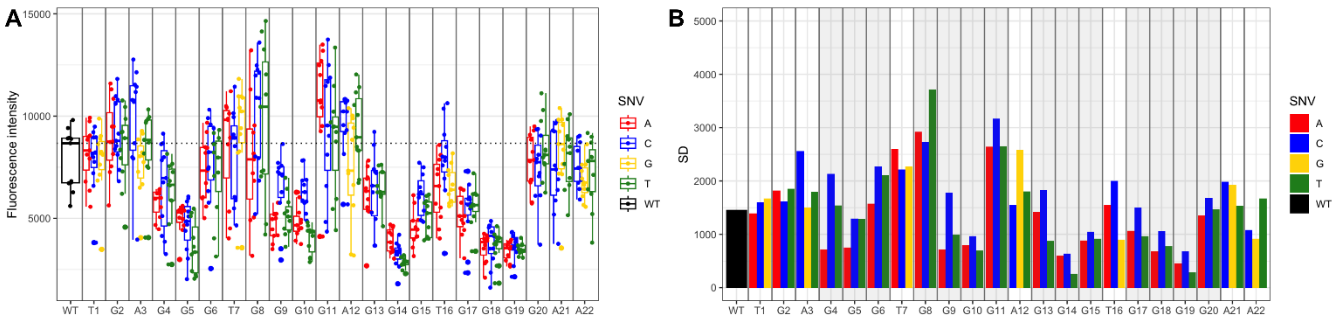

Figure S1. A) Plot of raw fluorescence intensities for individual probe sequences containing the indicated SNVs at each position of the MycG4. The horizontal dashed line indicates the median fluorescence intensity of Myc22 WT. (B) Plot showing the standard deviation of fluorescence intensities computed from probes containing the indicated SNVs.

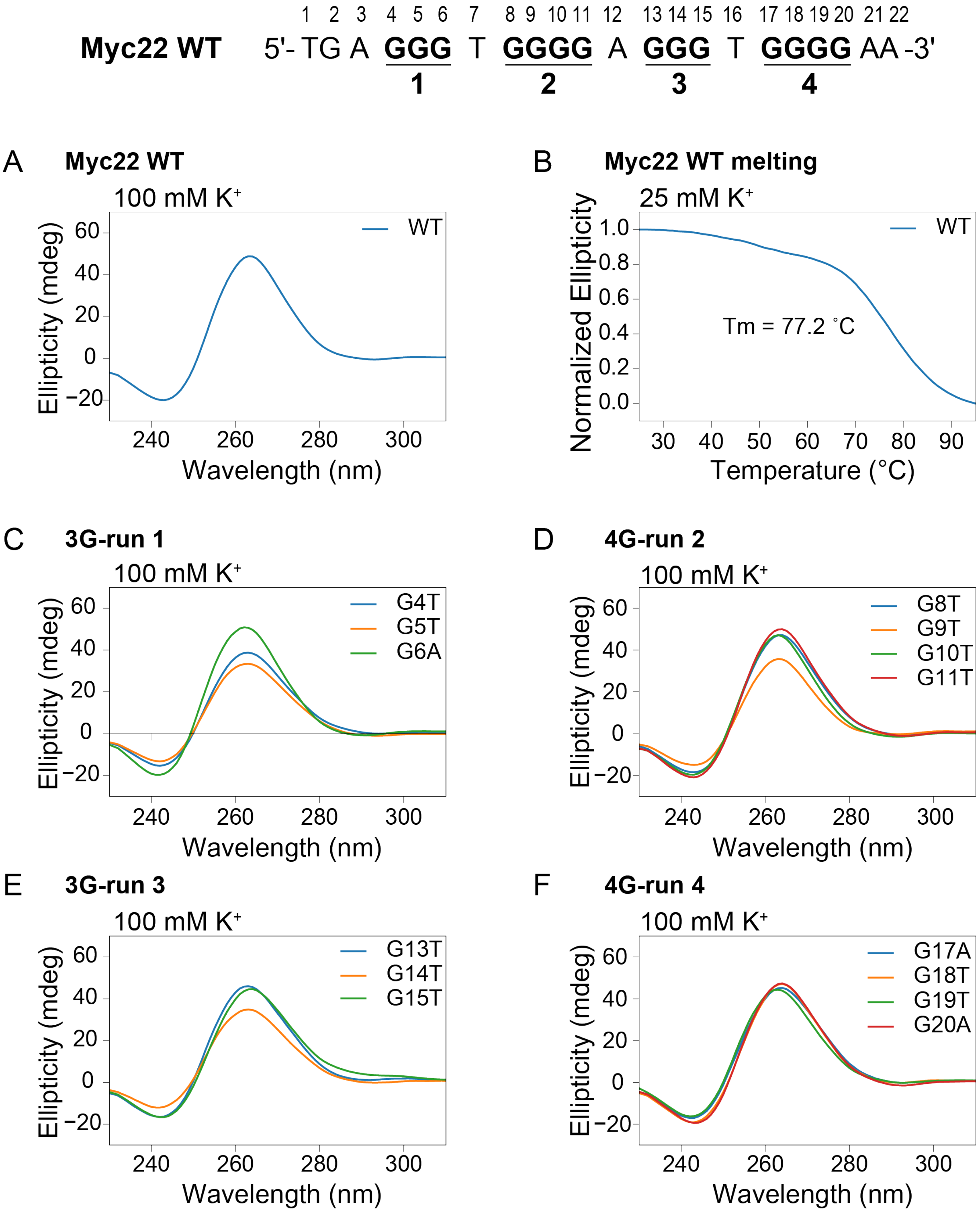

Figure S2. A) CD spectrum of Myc22 WT at 100 mM K^+. B) CD melting of Myc22 WT at 100 mM K^+. C) CD spectra of 3G-run 1 SNVs at 25 mM K^+ D) CD spectra of 4G-run 2 SNVs at 100 mM K^+ E) CD spectra of 3G-run 3 SNVs at 100 mM K^+ F) CD spectra of4G-run 4 SNVs at 100 mM K^+
